# From Genetic Maps to QTL Cloning: An Overview for Durum Wheat

**DOI:** 10.3390/plants10020315

**Published:** 2021-02-06

**Authors:** Pasqualina Colasuonno, Ilaria Marcotuli, Agata Gadaleta, Jose Miguel Soriano

**Affiliations:** 1 Department of Agricultural and Environmental Science, University of Bari ‘Aldo Moro’, Via G. Amendola 165/A, 70126 Bari, Italy; pattybiotec@yahoo.it (P.C.); ilaria.marcotuli@uniba.it (I.M.); 2 Sustainable Field Crops Programme, IRTA (Institute for Food and Agricultural Research and Technology), 25198 Lleida, Spain

**Keywords:** durum wheat, genetic mapping, QTL, GWAS, fine mapping, positional cloning, quality, abiotic stress, biotic stress

## Abstract

Durum wheat is one of the most important cultivated cereal crops, providing nutrients to humans and domestic animals. Durum breeding programs prioritize the improvement of its main agronomic traits; however, the majority of these traits involve complex characteristics with a quantitative inheritance (quantitative trait loci, QTL). This can be solved with the use of genetic maps, new molecular markers, phenotyping data of segregating populations, and increased accessibility to sequences from next-generation sequencing (NGS) technologies. This allows for high-density genetic maps to be developed for localizing candidate loci within a few Kb in a complex genome, such as durum wheat. Here, we review the identified QTL, fine mapping, and cloning of QTL or candidate genes involved in the main traits regarding the quality and biotic and abiotic stresses of durum wheat. The current knowledge on the used molecular markers, sequence data, and how they changed the development of genetic maps and the characterization of QTL is summarized. A deeper understanding of the trait architecture useful in accelerating durum wheat breeding programs is envisioned.

## 1. Introduction

The United Nations expects the world’s population to grow from seven to nine billion by 2050 (https://www.un.org/development/desa/en/news/population/world-population-prospects-2019.html; accessed on 25 January 2021). Crop production needs to satisfy future demands while facing extreme climate changes and hurdles in natural resources (such as water and soil) and disease management (https://www.fao.org; accessed on 24 July 2020). The main challenge faced by plant scientists in the 21st century is to increase the crop productivity per unit area and, at the same time, enhance sustainability and preserve biodiversity (European Plant Science Organization, https://epsoweb.org/; accessed on 24 July 2020). These have important implications on the breeding efforts and strategies for cereals, particularly wheat, which is a main source for food consumption worldwide.

Although common wheat (*Triticum aestivum* L.) is cultivated globally, durum wheat (*Triticum durum* Desf.) represents about 5% of the total wheat production, with almost 17 million ha worldwide and a global production of 38 million tons in 2019 (https://www.agr.gc.ca/; accessed on 25 January 2021), and is primarily cultivated in three different regions: the Mediterranean basin, the Northern Plains between the United States and Canada, and the desert areas of the south west of the United States and Mexico [1], characterized by a mix of variable and harsh environmental conditions (rainy weather, dry winters, and hot summers). Durum semolina is used for many products, including pasta, couscous, bread, and bulgur. Understanding the genetic basis of important agronomic traits in durum wheat is key for breeding programs. A significant yield increase was achieved with the introduction of semi-dwarf cultivars. Durum wheat germplasm developed by international Consultative Group on International Agricultural Research (CGIAR) centers (CIMMYT and ICARDA) were the most widely used by national programs worldwide. Besides these institutions, breeding programs in Italy were also very relevant from the beginning of the 20th century. According to Royo et al. [2] studying Spanish and Italian durum wheat cultivars from different periods, grain yield improvement was based on increasing the number of grains per unit area and harvest index, whereas grain weight did not change with the breeding process. In a similar work studying pasta quality, Subirà et al. [3] found that although protein content per grain was reduced, the yield increase produced by the new cultivars augmented the protein yield per ha. Other quality traits that increased significantly were the yellow index and gluten strength, obtaining very favorable allele combinations for high and low molecular weight glutenin subunits (HMW and LMW). The most important agronomic traits of cultivated plants are quantitative traits controlled by several genes across the genome and often influenced by the environmental conditions. The identification of major regions in the genome controlling these traits, quantitative trait loci (QTL), offers the opportunity to track them with molecular markers through molecular-assisted selection. Thus far, genetic maps and phenotypic data from segregating populations have allowed researchers to localize and map important genes, and to identify closely associated markers for marker-assisted selection (MAS) and, eventually, positional cloning [4,5]. However, the selection steps have been labor intensive and sometimes elusive, hindered by the development of extremely large mapping populations, non-specific molecular markers, large wheat genome size, and the lack of sequence information.

The identification of durum wheat QTL focuses on its main agronomic traits, including the protein grain content [6], high grain yield [7], disease resistance [8,9], and quality traits [10]. The studies used molecular markers such as simple sequence repeats (SSRs), expressed sequence tags (ESTs), and restriction fragment length polymorphism (RFLP) markers [11]. Standard mapping procedure through these DNA markers allowed for the identification of regions of several centiMorgans (cM) on the genetic maps, indicating the involvement of main genes. This non-specificity makes it difficult to find the key locus responsible for a specific trait, and the bystander effect of unwanted genomic regions is also difficult to control.

In the last decade, DNA sequencing technologies and applications including the discovery of new types of molecular markers have significantly improved plant breeding and assisted fine mapping procedures. With the completion of the Durum Wheat Genome Project [12], the scientific community examined the whole-genome sequences and all the data available from the studies regarding genes and QTL locations, the common variations found in genetic polymorphism sites, and other information that provides a large number of single nucleotide polymorphisms (SNPs) in the whole genome. Coupled with the rapid development of high-throughput genotypic technology, intense fine mapping analysis has been conducted in both a time- and cost-effective manner.

In the present review, we summarize the recent studies regarding the fine-map QTL, cloning QTL/genes, and identification of candidate genes for the main quality traits as well as biotic and abiotic stress resistance in recent years in durum wheat. The results presented can be very useful to program future studies and to identify major and stable QTL that can be considered to be cloned or further investigated by researchers for future applications in plant breeding programs.

In recent years, durum wheat research has undergone considerable expansion due to various agronomic, genetic, and commercial factors. The importance of durum wheat end-products in the food chain makes it crucial to maintain or increase durum wheat production under disease pressure and adverse climatic conditions but while preserving the grain quality. Thus, the grain quality and agronomic traits are also equally significant in determining the quality and yields of the processed products.

Here, we report the research into the improvement of wheat traits focused on the grain/flour quality of the breeding lines, environmental effects, disease resistance, development of evaluation methods, and processing for end uses [13].

## 2. Genotyping Tools

In the early 1990s, many agronomic complex traits were analyzed through polymorphic markers based on a hybridization method, such as restriction fragment length polymorphism (RFLP) markers [14], and polymerase chain reaction (PCR) techniques [15], such as random amplified polymorphic DNA (RAPD) [16], SSR [17,18], and amplified fragment length polymorphism (AFLP) markers [19].

The application of PCR led to the explosion of ESTs available in several plant databases, which have revolutionized current molecular biology and genetic approaches. In this revolutionary phase, DNA-based markers represent solid bases in plant breeding programs for their high rate of polymorphisms, numerous alleles for each locus, genome-wide distribution, and compliance to automation. However, the advances in high-throughput sequencing technologies provide new solutions for the development of high-density genetic maps, such as diversity array technology (DArT) [20], Genotyping-By-Sequencing (GBS) [21], and SNPs [22], and they were slowly replaced. These new technologies allow the identification of candidate genes within a few Kb in complex genomes. All of these sequencing applications have been a source of intense research. Here, we summarize some of the most recent methods and opportunities.

Due to the high number of SNPs in genomes compared with other DNA-based markers, high-throughput SNP discovery technology has been used more commonly for fine mapping studies. In particular, high-throughput sequencing technologies gave rise to millions of SNP markers [23], allowing the creation of DNA arrays, including those used for durum wheat such as the wheat 9K iSelect SNP array [24], the Illumina Wheat 90K iSelect SNP genotyping array [25], the Wheat 15K SNP array [26], the Axiom^®^ Wheat 660K SNP array, the Wheat 55K SNP array, the Axiom^®^ HD Wheat genotyping (820K) array [27], the Wheat Breeders’ 35K Axiom array [28], and the Wheat 50K Triticum TraitBreed array [29]. These arrays are efficient in the detection of polymorphisms in landraces and germplasms, increasing the availability of SNP markers [30], in particular the 820K array, derived from the exome capture technique of 43 wheat and wild species accessions, including elite cultivars, landraces, synthetic hexaploids, and wheat relatives.

Among these arrays, the high-density (90K) wheat SNP array developed by Wang et al. [25] represents the most used tool, including more than 8000 SNPs from durum cultivars [31]. The 90K SNP array was characterized by sequence information derived from 19 bread and 18 durum wheat lines, in addition to sequences from 28 [24], 24 [25], 8 [32], and 23 [33] wheat genotypes.

SNPs were widely used for the development of highly saturated genetic maps with 100,000 loci [5,34] for genetic diversity studies [35] and several SNP-based consensus wheat maps [31]. This intense application of SNP markers was mainly due to the dramatic reduction in sequencing costs, encouraging researchers to have a deep view of the target genomic regions by sequencing entire mapping populations [36]. Although the amount of SSR markers is the lowest compared to other SNP arrays, their combination has allowed analysis of the diversity in wheat, as demonstrated by Sajjad et al. [37], and fine mapping [38].

## 3. Linkage and Consensus Maps

Molecular markers have been widely used for the construction of linkage maps, representing the position of molecular markers along the chromosomes measured in genetic distance, based on the recombination events between individuals. The different applications of linkage maps in plant breeding are crucial for the identification of the associations of molecular markers with traits of interest for gene discovery, comparative genomics between different species, and physical mapping to facilitate genome assembly. Linkage maps have been developed for most crop species, from fruit trees to vegetables and cereals.

The first linkage map in durum wheat was developed by Blanco et al. [39]. This map was mainly constructed using restriction fragment length polymorphism (RFLP) markers. Since 2000, throughout the decade, other linkage maps, including inter-specific maps resulting from the cross between durum wheat and wild emmer, were developed. They were constructed mainly with SSR or the newly developed diversity arrays technology (DArT) markers developed by Diversity Arrays Technology Pty Ltd. (Canberra, Australia) with the microarray technology platform [20]. Through hybridization-based methods, diversity arrays detect single base pair changes (SNPs) [40]. DArTs became highly used markers as they provide extensive genome coverage, and they are obtained through a low-cost marker system. Table 1 summarizes the main durum wheat linkage maps reported in the literature.

Based on the high coverage reported utilizing the newly developed DArT markers, new consensus maps of durum wheat were developed by Marone et al. [41] and Maccaferri et al. [4]. These consensus maps integrate different linkage maps from several mapping populations containing common markers that act as anchor markers between the maps. These maps provide higher marker coverage and serve to correct incongruences between individual maps [42], thus improving the fine mapping of genes of interest. Recently, advances in next-generation sequencing (NGS) technologies have reduced the costs of DNA sequencing, making it feasible to genotype base on sequence data. These advances fostered the development of high-throughput SNPs platforms [25,43] and the development of DArTseq technology based on genotyping by sequencing (GBS) (https://www.diversityarrays.com/; accessed on 24 July 2020). With these high-throughput platforms, Maccaferri et al. [31] constructed a second-generation consensus map for durum wheat.

Three consensus maps have been developed for durum wheat in the last decade. The consensus map developed by Marone et al. [41] integrated six linkage maps, with a marker coverage of 0.15 to 0.46 markers/cM, containing a total of 1898 loci, mainly DArTs (1185), covering a total distance of 3059 cM with a mean coverage of 0.62 markers/cM. A total of 650 markers were shared by at least two individual linkage maps. Maccaferri et al. [4] also developed a consensus map integrating six linkage maps as a framework, including 598 markers, mainly SSRs (295) and DArTs (281), and 1977 markers were interpolated from eight other mapping populations. The consensus map contained a total of 2575 markers covering 2463 cM, which represents a coverage of 1.04 markers/cM and is a significant improvement from the original maps, which ranged from 0.08 to 0.48 markers/cM. More recently, with the development of SNP arrays, Maccaferri et al. [31] constructed a new consensus map integrating this technology. A total of 13 biparental mapping populations were used, and the final map included 30,144 markers, mainly SNPs, from the array developed by Wang et al. [25] spanning 2631 cM. The marker coverage increased from 4.83 markers/cM in the most saturated linkage map (Svevo × Zavitan) to 11.45 markers/cM in the consensus map.

## 4. Traits and QTL Analysis

### 4.1. Quality

Durum grains, a storehouse of nutritional elements for the human diet, contain starch (70.2%), proteins (12.2%), lipids (1.9%), fiber (1.6%), and minerals (1.6%), with varying water content [79]. In addition, the kernels comprise high carbohydrate and antioxidant (such as carotenoid pigments) contents, together with high vitamin, potassium, calcium, sodium, and magnesium levels.

The kernel quality is of paramount importance in the end-product quality of commercial wheat varieties, determining the type of products that can be produced. Thus, it is possible to distinguish a commercial value in the beginning of wheat production and a technological value linked to the worldwide market requirements for end-product uses.

For the commercial value, the wheat quality is primarily evaluated through the milling rate, which consists of the quantity of wheat flour derived from 100 kg of seeds. This rate aims to obtain the maximum quantity of flour, analyzing the seed size, thousand-kernel weight, ash mass (or mineral content), and the percentage of seed defects, such as pre-germination, small or white seeds, and pathologic darkness of the grain. For the technological value, wheat quality is evaluated for the strength of the flour protein when mixed with water to make dough (e.g., hardness, quantity and quality of the proteins, and rheological parameters), the quantity of water required for workable dough, and the flour color (e.g., yellow color). The protein quantity and quality influence the characteristics of the end wheat products: for example, a high protein content brings major water absorbance, increasing the productivity rate and the shelf life of the final products. Several investigations [54,80,81,82,83,84,85] indicated that factors influencing the protein concentration in cultivated and wild wheat include quantitative trait loci (QTL) located almost on all chromosomes.

Carotenoid pigments have an enormous importance for the nutritional value for human health and affect the wheat flour color. The antioxidant activity of carotenoids, together with protein, increases the nutritional and technological characteristics of flour [86,87]. An example of flour with a high carotenoid content is whole wheat flour, which also contains high levels of fiber (β-glucan and arabinoxylan), vitamins, and antioxidant molecules such as tocopherols and flavonoids, which are also considered to be kernel components that are important for quality.

Another important trait related to wheat quality is the starch content (75% of the weight of the mature grain), which consists of two types: amylose and amylopectin. In fact, the water absorption of the dough is connected to the starch content [88,89]. High starch content changes the functional properties of the flour, such as the gelatinization, pasta characteristics, and baking applications [90]. This aspect is important in the production of baked foods, such as cakes and some types of breads, providing a tender status to the final products [79].

### 4.2. Biotic Stress

Major breeding programs are also focused on improving biotic stress resistance because of the severe damage to production worldwide. These kinds of stresses are generated by living organisms (fungi, bacteria, insects, etc.) causing diseases such as rust, powdery mildew, *Fusarium graminearum*, and various viruses.

Amongst the pathogens damaging durum wheat crops at the leaf and stem levels, rust pathogens are the most prevalent. This includes leaf rust (*Puccinia triticina*), stem rust (*P. graminis tritici*), and stripe rust (*P. striiformis*), causing losses of 15–20% worldwide [91]. To date, over 77 genes conferring resistance to leaf rust (Lr) have been characterized and localized to specific wheat chromosomes [92]. Durum wheat has been historically more resistant to leaf rust compared with bread wheat; however, this kind of resistance could evolve into a rapid breakdown due to the birth of new virulent races.

Powdery mildew (*Blumeria graminis* f. sp. *tritici*) is recognized as a disease of wheat of high economic importance, especially in warm climate areas where the productivity is high. Therefore, one of the most important purposes of breeding programs is to make resistant durum wheat. To achieve this goal, several studies, including a careful analysis of the environmental conditions that can influence the host–pathogen interactions, a study of the genetic and molecular interactions between the host and pathogen, and the search for new sources of resistance to be transferred in varieties, are required [93].

Fusarium head blight (FHB) is one of the most common and harmful diseases of durum wheat in the world. The incidence and severity of FHB depends on the weather, the areas, and the varieties used. This pathogen has caused consequential production losses together with damage to the quality of grain and the presence of mycotoxins. The presence of *Fusarium* toxins (deoxynivalenol, DON) in wheat represents a serious hygienic–sanitary problem [30,94].

### 4.3. Abiotic Stress

In the Mediterranean Basin, durum wheat is cultivated under variable environmental conditions. In rainfed agricultural environments, drought stress critically constrains the crop yield. It is particularly challenging for breeders under the current unpredictable climate change to stretch the adaptability and performance stability of their cultivars. In the Mediterranean, the environment is responsible for as much as 98% of bread and durum wheat yield variations [95].

Wheat yield is dependent on the grain number per unit area and grain weight. With climate change, yield reduction is led by a significant decrease in one or both yield components. The grain number, in turn, may be split into spikes per unit area and grains per spike. The yield components are sequentially determined and are counter-dependent. Reductions in the grain number per unit land area due to an increase in temperature have been widely reported, as has a reduction in the grain weight, which depends on the environmental conditions before flowering and during grain filling [96,97]. Although drought stress in the pre-flowering period can influence the grain weight [98], the grain filling period is considered critical for the final grain weight [99]. In Mediterranean environments, water becomes mostly limiting after anthesis, typically being accompanied by high temperatures, which causes a reduction in the yield potential of approximately 50% [100]. The main impact of drought stress after anthesis is to reduce the grain setting, size, and weight [101].

Breeding for adaptation to drought is extremely challenging due to the complexity of the target environments as well as the stress-adaptive mechanisms adopted by plants to withstand and mitigate the negative effects of water deficit [102]. The incomplete knowledge of the physiological and genetic basis of drought resistance [103] as well as insufficient consideration of drought environments when defining target traits for stress resistance [104] may explain the low yield improvements observed in wheat grown in dry regions [105].

Understanding the physiological mechanisms associated with drought resistance and the genetics underlying them may provide new strategies for engineering varieties resilient to drought, which is essential for wheat breeding [106,107]. The crop traits to be considered as selection targets under drought conditions must be genetically correlated with the yield and should have a greater heritability than the yield itself [99,108]. Among them, early vigor, leaf area duration, crop water status, radiation use efficiency, and root architecture have been identified as being associated with the yield under rainfed conditions (reviewed in [109]).

Another important constraint in crop production is heat stress. This is tightly associated with drought in the Mediterranean region as it is of particular importance when it occurs between heading and maturity, affecting the flowering and grain filling, thus reducing the grain yield [110]. Heat stress in plants produces a wide range of effects, affecting their physiology and altering their gene regulation, causing a decrease in the synthesis of proteins [111]. The ability of plants to cope with high temperatures is called thermotolerance and can be measured through the increase in the fluidity of the cellular membrane, causing the escape of electrolytes to the extracellular medium [112].

Soil salinity is considered an important limiting factor for crop production in arid and semi-arid regions. Although related to drought stress, salinity affects plant growth during the whole cycle of plant development [113]. The last factor to be considered as abiotic stress is cold and/or frost tolerance, although growing winter wheat in regions such as Central Europe or North America substantially increases the yield potential [114].

### 4.4. QTL Mapping

Classically, QTL mapping has been performed predominantly in populations derived from two parents (biparental) with different phenotypic performances. The success in detecting QTL depends on the marker density, population size, and the heritability of the trait. With durum wheat, a large number of QTL studies have been performed to investigate yield performance, biotic and abiotic stresses, phenology, and quality.

**Figure 1:**
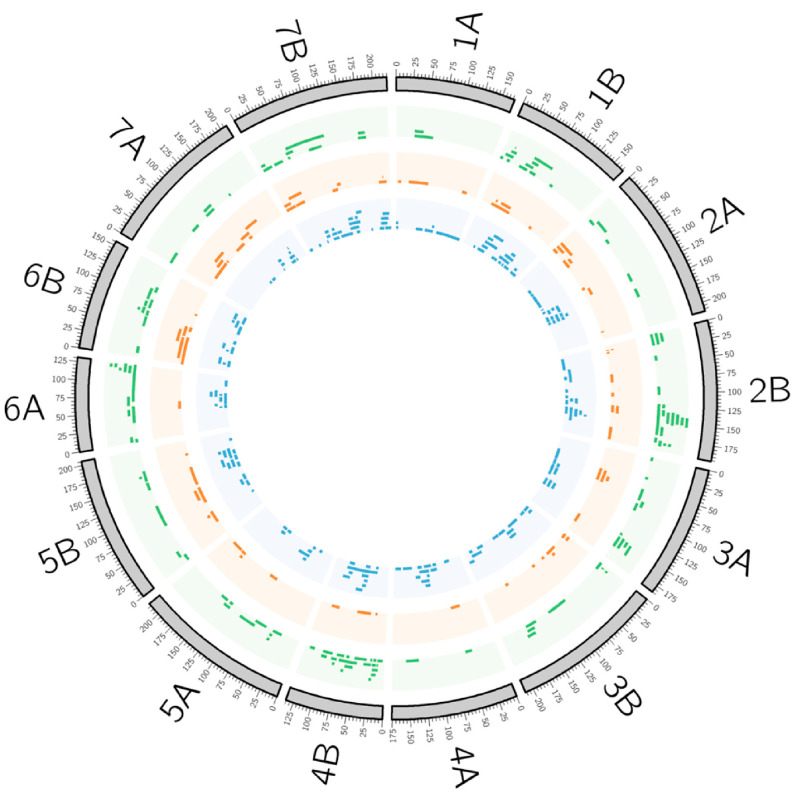
Quantitative trait loci (QTL) projection onto the consensus map developed by Maccaferri et al. [31]. Color code: green, abiotic QTL; orange, biotic QTL; blue, quality QTL.

The grain yield and phenology are traits much studied in wheat, as reviewed in Soriano et al. [115]; thus, the present review focuses on the traits involved in abiotic and biotic stress resistance and grain quality. To synthesize all the QTL in the same linkage map to obtain an overview of the QTL distribution, the durum wheat consensus map developed by Maccaferri et al. [31] was used for QTL projection based on the homothetic approach described by Chardon et al. [116]. A total of 45 QTL studies—six corresponding to abiotic stress tolerance, 24 to biotic stress resistance, and 15 to quality traits—were revised (Table 2 and Table S1), comprising 368 QTL, from which 127 corresponded to traits related to abiotic stress, 71 to biotic stresses, and 171 to quality-related traits (Figure 1).

### 4.5. Genome-Wide Association Studies

Association mapping or genome-wide association study (GWAS) is a complementary approach to dissect the genetic basis of complex traits, providing broader allelic coverage and offering higher mapping resolution. This is based on linkage disequilibrium (LD), defined as the non-random association of alleles at different loci, and is used to detect the relationship between phenotypic variation and genetic polymorphisms [117]. It is important, however, to differentiate the LD due to physical linkages from LD due to the population structure, which can be caused by selection, genetic drift, and species-dependent characteristics, such as the mating system. Germplasm collections characterized by medium to high LD levels are suitable for the identification of chromosome regions harboring genes/QTL controlling agronomic traits in wheat [118]. The main differences between biparental QTL mapping and GWAS are shown in Table 3. Many studies have been conducted in durum wheat to investigate the genetic basis of yield and yield components [115,119,120], crop phenology [115], or biomass [115]. Here, we summarize a total of 19 GWAS studies that identified marker–trait associations (MTAs) involved in abiotic (4) and biotic (8) stress and grain quality (7) (Table 2).

A summary of the total number of QTL and MTAs reported in durum wheat with the analyzed traits is shown in Table 4. Our review included ten traits related to abiotic stress, nine related to biotic stress, and nineteen related to quality traits.

### 4.6. QTL Meta-Analysis

As reported in Table 2, hundreds of QTL have been reported for the different traits considered. One way to synthesize this information is the QTL meta-analysis developed by Goffinet and Gerber [154]. The aim of QTL meta-analysis is the identification of genome regions repeatedly involved in trait variation to narrow down the QTL supporting intervals and make them useful for breeding and enabling the identification of candidate genes. The process involves several steps: the construction of a consensus map integrating different types of markers or the use of a reference map for the species; the projection of the initial QTL on the consensus maps; or the estimation of consensus regions harboring different QTL or meta-QTL.

To select the most appropriate meta-QTL for breeding purposes or candidate gene isolation, Löffler et al. [155] established three criteria that the meta-QTL must meet: (1) a small supporting interval, (2) integrating a high number of original QTL, and (3) a high effect of the phenotypic variance explained by the original QTL.

QTL meta-analysis has been performed in both bread wheat and durum wheat for different traits such as grain yield [112], crop phenology [112,156], disease resistance [155,157,158,159], plant height [160], grain-related traits [161,162], root-related traits [118,163,164], and sprouting tolerance and dormancy [165].

The use of consensus maps integrating the most common molecular markers and the identification of consensus QTL regions among different mapping populations from different parental sources will help breeders to select the most appropriate plant material for the development of new cultivars. Redefining (and shortening) QTL regions by QTL meta-analysis will help with the identification of candidate genes for accelerating the breeding process. The use of the recent release genome sequence of durum wheat [12] will allow the identification of the physical regions of QTL of interest to abord map-based cloning strategies.

## 5. Innovative Experimental Designs for Enhanced Gene Discovery

### 5.1. Bulked Segregant Analysis by Sequencing (BSAseq)

The analysis of transcriptome through SNP arrays allowed the combination of bulked segregant analysis (BSA) and RNA sequencing to fine-map small proportions of a wheat genome involved in the traits of interest [61]. This strategy has been successfully used for identifying candidate genes and gene cloning purposes in maize [166] and bread wheat [167,168,169]. In durum wheat, BSAseq was used to refine the species cytoplasm-specific (*scs*) locus [170]. The limitation of BSAseq in tetraploid wheat could be due to the presence of co-expressed homoeologous genes and the absence of a good reference sequence for mapping, which was completed last year [12].

### 5.2. Development of New Populations

As described above, the main limits of biparental QTL analysis rely on the low number of recombinant events, the limited number of polymorphic markers, and the reduced availability of genetic diversity. To overcome these drawbacks, in recent years, new breeding programs and new plant populations have been proposed, increasing the statistical power and the association between markers and traits and allowing the identification of key genes involved in the phenotypic variation [171]. These include the multiparent advanced generation intercross (MAGIC) and the nested association mapping (NAM) populations, both based on multiple founders.

In particular, the MAGIC populations are generated through inter-crossing several parental lines (eight or more) through two-way, four-way, and eight-way crosses and subsequent self-fertilization to generate recombinant inbred lines (RILs). Compared to traditional biparental populations, the use of more founders in the initial crosses allowed an increase in the recombination, consequently improving the mapping resolution and allowing greater allelic diversity. The MAGIC design was successfully realized in several crops for fine genetic mapping; however, a slow uptake has been observed in durum wheat, where a four-parent durum wheat population was produced [172]. A successful four-way durum wheat MAGIC population was developed by Milner et al. [173] for the first time by crossing four elite cultivars from different origins. The population was used to study important quantitative traits such as heading and maturity date, plant height, and grain yield. The results reported based on the analysis based on founder haplotype probabilities proved to be more efficient in QTL mapping in this multiparent population as compared to a conventional bi-allelic assay. In the present review, we reported studies on 40 traits that can help breeders and researchers in the choice of the most useful genotypes carrying major QTL for the production of new MAGIC populations in durum wheat.

The NAM populations were developed through crossing several founders with the same reference line to generate a series of “interconnected” segregating inbred families. The advantage of this population is represented by the possibility of incorporating a high number of alleles in one species gene pool, accurate QTL effects and positions, and overcoming the limits of linkage analysis and association mapping approaches [174,175].

The NAM design for QTL mapping was developed in maize [176,177] and later applied to other crops including soybean [178], sorghum [179], barley [180], bread wheat [181], and durum wheat [182,183,184].

Germplasm resources as landrace collections could be of interest for the development of new mapping populations following the approaches of MAGIC and NAM to incorporate allelic diversity. As reported by Soriano et al. [185], Mediterranean landraces represent an important group of genetic resources due to their good adaptation, huge genetic diversity, resilience to abiotic and abiotic stresses, and their differences in yield formation strategies [186].

### 5.3. Candidate Gene: A New Approach for Studying Quantitative Trait Loci

One of the main objectives of molecular genetics is to identify and isolate genes controlling important traits. Three main approaches lead to the cloning of genes of interest: positional cloning [187], insertional mutagenesis [188], and candidate genes (CGs) [189,190]. The last strategy has been successfully used after the assumptions regarding the biological function of the gene of interest. It started from sequenced genes of known function that could correspond to major loci (Mendelian trait loci, MTLs, or quantitative trait loci, QTL). CGs may be structural genes or genes involved in the regulation of a metabolic pathway [189]. The hypothesis is that a molecular gene polymorphism is related to the phenotypic variation.

The CG approach is based on three chronological steps: (1) choice of CGs, established through molecular and functional studies or based on linkage data; (2) molecular polymorphism in the CG to localize it on a genetic map or to calculate statistical correlations between CG polymorphisms and phenotypic variation in a set of unrelated individuals; and (3) complementary experiments (if map co-segregation and/or statistical correlation were found) to confirm the real association of the CG in the trait variation [191]. The CG approach is more adapted to QTL characterization than positional cloning or insertional mutagenesis due to the multiplicity of genes involved in the trait, their partial effect, and indefinite genetic map positions.

The CG approach has been used with success in durum wheat for many traits such as disease resistance [34,192], carotenoid content (as reported in [87]), protein content [85], fiber (as reported in [79,193]), and salinity stress [194].

Thanks to the recent release of durum wheat genome [12] and gene expression analysis databases as the wheat expression browser [195] (http://www.wheat-expression.com/; accessed on 25 January 2021), new advancements in CG identifications can be obtained through the application of new computational methods developed to prioritize CGs in QTL before functional studies. The identification of the gene *TdHMA3-B1* responsible for variation in cadmium accumulation in the grain is a successful example of the use of the genome sequence data together with the genetic variation reported in germplasm collections as the global durum panel, as reported in [12].

Moyers [196] developed a computational approach, “Camoco”, that integrates loci identified by GWAS with functional information derived from gene co-expression networks. More recently, Lin et al. [197] developed an algorithm, “QTG-Finder2”, for prioritizing QTL causal genes in plants and validated *Sorghum bicolor* and *Setaria viridis* models.

## 6. Gene Editing in Durum Wheat

Genome editing is a new biotechnology allowing specific manipulation of a target genomic sequence, which has the potential to improve wheat performance and supersede traditional methods in plant breeding.

This technology in wheat is made more problematic due to the complexity of the wheat genome and the difficulty in genetic transformation. Despite this, recent progress in plant genome editing has been reported, above all, for bread wheat. One of the first papers using the CRISPR/Cas9 system in wheat was reported in 2014 by [198], who reported a mutation of three homoeoalleles *TaMLO* in hexaploid wheat for a gene that confers heritable resistance to powdery mildew.

The scientific community have concentrated their efforts on resistance to pathogens such as powdery mildew [198]; leaf rust; the analysis of a pathogenesis-related protein 1 Gene, *TcLr19PR1*, involved in resistance against leaf rust as reported by [199]; stripe rust with the resistance gene *Yr10*, which encodes an evolutionarily conserved and unique CC-NBS-LRR sequence in wheat [200]; sharp eyespot disease [201]; and resistance to *Bipolaris sorokiniana* with an *R2R3* MYB transcription factor in wheat, *TaPIMP1* [202].

In addition, yield was the second most studied trait; the genes *TaGASR7*, *TaGW2*, and *TaLOX2* in hexaploid wheat and only *TdGASR7* in tetraploid durum wheat were edited because they were found to be associated with grain yield components [203,204,205]. However, the research in this field is rapidly increasing. Recently, two independent studies [206,207] reported CRISPR/Cas9 editing of the α-Amylase/Trypsin inhibitor (ATI) genes, reducing the allergen proteins in durum wheat. The ATI subunits WTAI-CM3 and WTAI-CM16 from the durum wheat cultivar Svevo were edited to produce lines with reduced allergens. The authors obtained durum wheat lines with stable and heritable mutations in these genes. These lines will be of special interest for durum wheat breeding programs for the introgression of the alleles in other elite cultivars.

The recent advance in high-quality reference genomes for durum wheat along with new efficient genetic transformation strategies will accelerate the application of genome editing technology.

## 7. From Classical Breeding to Genomic Selection

The selection methods used in wheat breeding programs have deeply evolved with time. Classical breeding based on the phenotypic selection of a trait was the main approach used by breeders to increase crop productivity during the 20th century [208]. This technique implies the selection of varieties carrying the desired characteristics for the target trait, usually morphological or visual features such as the yield, yield components, or disease resistance. Despite the large improvements in genetic gains for yield and quality obtained in bread [209,210] and durum wheat [3,211], this process takes several years to finally obtain a commercial variety and has some limitations, especially when target traits are highly dependent on the environment due to low heritability [212].

The development of molecular biology allowed the use of markers based on the sequence or polymorphisms in DNA for the identification of traits enhancing agronomic performance in the earlier stages of development. Marker-assisted selection (MAS) resulted in an important advantage integrated with traditional breeding methods to enhance the efficiency of cultivar development. However, MAS depends on the genetic linkage of traits with markers, and typically, only genetic loci with major effects are exploitable in this way [213]. Since many agronomic traits present a multigenic quantitative nature and the effect of the environment on them needs to be assessed, MAS cannot replace traditional breeding methods for these traits, particularly in later-generation screening and cultivar evaluation.

The availability of high-density, low-cost marker genotyping platforms has enabled a change in plant breeding by making genomic prediction and selection feasible. Genomic selection (GS) refers to the selection of genotypes using genomic information on a genome-wide scale to make selections [214]. Genomic selection uses genome-wide markers to estimate the effects of all genes or chromosome positions simultaneously to predict the breeding values of progeny, which are used for the selection of individuals without costly phenotyping, saving money and time and increasing the accuracy of selection [214].

Different studies in durum wheat applied this technology for predicting the grain yield, quality traits, and disease resistance. Fiedler et al. [215] found prediction accuracies from 0.27 to 0.66 for different grain and semolina quality traits using 1184 breeding lines from the North Dakota State University (NDSU) durum wheat breeding program. Haile et al. [216] studied the potential of single- and multi-trait genomic prediction models on grain yield and quality traits using a breeding panel comprising 170 cultivars and advanced lines as well as a doubled haploid population.

The accuracy found by these authors ranged from 0.5 to 0.8 for single traits, increasing in multi-traits for grain yield. Zaim et al. [217] used four populations to develop genomic prediction models for grain yield under drought conditions. These authors found that including QTL information from the populations increased the prediction accuracies by 0.06 to 0.12 points. In addition to grain yield and quality traits, disease resistance is another target for GS. In this sense, Moreno-Amores et al. [218] assessed the prediction ability for fusarium head blight (FHB) using plant height and heading date as covariates as they are influenced negatively by FHB.

## 8. Speed Breeding

The development of speed breeding protocols can be considered as a useful approach to help in the development of new mapping populations and to advance the first generations in the breeding programs, which, being assisted by molecular markers, will save time for breeders, reducing the length of the breeding cycles and selecting the best genotypes. The technique implies the use of extended photoperiods in a controlled environment. For durum wheat, up to six generations in a year have been achieved [219]. These protocols have been adapted for multi-trait phenotyping in durum wheat, as described by Alahmad et al. [220] for the rapid selection of generations and the characterization of breeding lines anytime during the year.

## 9. Future Prospects

To meet the food needs for the future, farmers must increase crop yields considering the climate and environment changes. Information gained from sequenced genomes in related species and in durum wheat, together with studies of fine mapping and QTL cloning, allows the identification of a high number of molecular markers, key genes, quantitative trait loci, and networks, which will lead to higher yielding crops.

The decreasing cost of NGS technologies and the huge availability of sequence data on web databases allow for drastic reductions in the time required to identify candidate genes. Thus, projects that previously required more than 10 years for fine mapping, QTL cloning, and candidate gene identification could now be completed in 1–2 years for more simple traits.

## Figures and Tables

**Table 1:** Durum wheat linkage maps.

Cross	Type	*N* Progenies	*N* Markers	Marker Type	Distance (cM)	Reference
Messapia × MG4343	RIL	65	213	RFLP, biochemical	1352	[39]
Messapia × MG4343	RIL	65	292	SSR, RFLP, biochemical	2034	[43]
Messapia × MG4343	RIL	65	293	AFLP, RFLP, biochemical	2063	[44]
Jennah Khetifa × Cham 1	RIL	110	306	AFLP, SSP, SSR	3598	[45]
Messapia × MG4343	RIL	65	458	AFLP, RFLP, SSR, biochemical, morphological	1352	[46]
Omrabi 5 × 600545	RIL	114	295	AFLP, SSP, SSR	2289	[47]
Strongfield × Blackbird	DH	85	424	SSR	2052	[48]
W9262-260D3 × Kofa	DH	155	194	DArT, SSR	1400	[49]
Colosseo × Lloyd	RIL	176	554	DArT, SSR	2022	[50]
Langdon × G1816	RIL	152	669	DArT, SSR	2317	[51]
Kofa × UC1113	RIL	93	269	SNP, SSR	2140	[52]
Svevo × Ciccio	RIL	120	522	SSR	1605	[11]
Creso × Pedroso	RIL	123	456	DArT, SSR	1800	[53]
DT695 × Strongfield	DH	185	345	DArT, SSR	1474	[54]
Latino × Primadur	RIL	121	454	DArT, SSR	1172	[10]
Neodur × Cirillo	RIL	146	414	DArT, SSR, STS	1917	[55]
Svevo × Ciccio	RIL	120	833	DArT, SSR, morphological	1716	[7]
DS × Td161	BC	134	368	AFLP, SSR	1921	[56]
Floradur × Td161	BC	129	248	AFLP, SSR	1414	[56]
Helidur × Td161	BC	126	239	AFLP, SSR	1515	[56]
BGRC3487/2 × DT735	RIL	160	948	DArT, SSR	1089	[57]
Rugby × Maier	DH	105	228	DArT, SSR	801	[6]
PDW233 × Bhalegaon 4	RIL	140	245	SSR, ISSR, TRAP, SCAR, STS, biochemical	2773	[58]
Gerizim #36 × Helidur	RIL	103	421	AFLP, SSR	1808	[59]
Wollaroi × Bansi	RIL	92	799	DArT, SSR	3859	[60]
Svevo × Ciccio	RIL	120	5670	DArT, SNP, SSR	1774	[5]
Ben × PI41025	RIL	200	2593	SNP, SSR, STS	2444	[61]
Simeto × Molise Colli	RIL	136	9040	SNP	2879	[62]
Latino × MG5323	RIL	110	10840	SNP, SSR	2363	[63]
Kofa × Svevo	RIL	249	311	SNP, SSR	1259	[4]
Gallareta × Demetra	DH	127	147	DArT, SSR	1017	[4]
DT707 × DT696	DH	127	136	DArT, SSR	861	[4]
DT712 × Blackbird	DH	89	392	SSR	1848	[4]
Lebsock × PI94749	DH	146	240	SSR, biochemical	1463	[4]
PDW1216 × MvTD10-98	RIL	182	440	SSR	984	[4]
W9292-260D3 × Kofa	DH	155	3712	SNP, SSR	1685	[31]
Svevo × Zavitan	RIL	140	10911	SNP	2258	[31]
Simeto × Levante	RIL	180	5978	DArT, SNP, SSR	2185	[31]
Mohawk × Cocorit69	RIL	81	5554	SNP	2013	[31]
Meridiano × Claudio	RIL	180	5970	DArT, SNP, SSR	2239	[31]
Colosseo × Lloyd	RIL	176	7946	DArT, SNP, SSR	2064	[31]
Kunduru-1149 × Cham 1	RIL	141	395	AFLP, SSP, SSR	4854	[64]
02-5B-318 × Saragolla	RIL	135	4366	SNP	4227	[30]
Karur × DBC-480	RIL	110	1609	DArTseq, SSR	2806	[65]
Durobonus × DBC-480	RIL	100	1052	DArTseq, SSR	1781	[65]
SZD1029K × DBC-480	RIL	100	1006	DArTseq, SSR	2219	[65]
Bairds × Atred#1	RIL	137	1150	SNP, SSR	2639	[66]
W9262-260D3 × Kofa	DH	155	4227	SNP	2282	[67]
Duilio × Avonlea	RIL	134	5444	SNP	1962	[68]
Ofanto × Cappelli	RIL	98	9267	DArT, DArTseq, SSR	2119	[69]
Joppa × 10Ae564	RIL	205	5216	SNP	3428	[70]
DT707 × DT696	DH	127	2943	SNP	1808	[71]
Strongfield × Blackbird	DH	85	9568	SNP	2763	[71]
Svevo × Y12-3	RIL	208	4166	SNP	2169	[72]
Faraj × Gidara 2	RIL	143	2067	SNP	2578	[73]
Tunisian 108 × Ben	BIL	178	329	DArT, SSR	1888	[74]
Zardak × Iran249	RIL	118	6195	SNP, SSR	2884	[75]
Rusty × PI193883	RIL	190	9346	SNP, SSR	2440	[76]
Rusty × PI192051-1	RIL	180	1138	SNP	1436	[77]
Rusty × PI 387336	RIL	200	2894	SNP	2858	[78]
Rusty × PI 387696	RIL	200	2059	SNP	2724	[78]
Rusty × PI 466979	RIL	200	3692	SNP	2776	[78]
Rusty × Iumillo	RIL	200	2911	SNP	2968	[78]

BC: backcross; DH: double haploid; RIL: recombinant inbred line; AFLP: amplified fragment length polymorphism; DArT: diversity array technology; ISSR: inter simple sequence repeat; RFLP: restriction fragment length polymorphism; SCAR: sequence characterized amplified region; SNP: single nucleotide polymorphism; SSR: simple sequence repeat; STS: sequence-tagged sites; TRAP: target region amplification polymorphism.

**Table 2:** Summary of quantitative trait loci (QTL) reviewed in the present work.

Reference	Cross	Type	*N* Genotypes	N QTL	Traits
**Abiotic stress**					
[121]	Langdon × G18-16	RIL	156	31	CIR, OP, CC, FLRI
[122]	Kofa × Svevo	RIL	247	12	PDL, SPAD, NDVI
[123]	Omrabi5 × Belikh2	RIL	114	6	CL, RRT
[124]	Colosseo × Lloyd	RIL	176	28	RRT
[124]	Meridiano × Caludio	RIL	181	32	RRT
[125]	Simeto × Mollise Colli	RIL	136	18	RRT
[126]	Elite cultivars	GWAS	57	4	RRT
[127]	Elite cultivars	GWAS	183	2	RRT
[124]	Elite cultivars	GWAS	183	31	RRT
[128]	UNIBO-DP	GWAS	248	73	DB, NDVI, SPAD
**Biotic stress**					
[48]	Strongfield × Blackbird	DH	85	2	FHB
[129]	LDN × LDN-Dic7A	RIL	118	1	FHB
[8]	Colosseo × Lloyd	RIL	176	1	LR
[119]	Meridiano × Claudio	RIL	181	1	SBCMV
[56]	DS × Td161	BC	134	1	FHB
[56]	Floradur × Td161	BC	129	3	FHB
[56]	Helidur × Td161	BC	126	1	FHB
[130]	Kristal × Sebatel	RIL	85	7	SR
[131]	Simeto × Levante	RIL	180	7	SBCMV
[57]	BGRC3487 × 2 * DT735	RIL	160	2	FHB
[55]	Neodur × Cirillo	RIL	146	2	SBCMV
[60]	Wollaroi × Bansi	RIL	92	2	YR
[59]	Gerizim × Helidur	RIL	103	1	FHB
[132]	Langdon × G18-16	RIL	157	4	PM
[63]	Latino × MG5323	RIL	110	3	LR
[133]	Ben × PI41025	RIL	200	3	FHB
[94]	Sumai-3 × Saragolla	RIL	135	11	FHB
[65]	Karur × DBC-480	RIL	111	1	FHB
[134]	Strongfield × Blackbird	DH	90	2	LS
[135]	Kofa × W9262-260D3	DH	155	1	YR
[70]	Joppa × 10Ae564	RIL	205	3	FHB
[77]	Rusty × PI 192051-1	RIL	180	5	LR
[74]	Ben × Tunisian 108	BIL	171	3	FHB
[136]	Greenshank × AC Avonlea	DH	132	4	CP
[137]	Different sources	GWAS	323	3	FHB
[138]	Elite cultivars	GWAS	183	8	SR
[139]	Worldwide collection	GWAS	496	50	LR
[140]	Ethiopian landraces	GWAS	318	20	STB
[141]	Elite cultivars	GWAS	250	16	YR
[135]	Elite cultivars	GWAS	92	1	YR
[142]	Tetraploid panel	GWAS	230	37	SR
[143]	Spring lines	GWAS	228	7	FHB
**Quality**					
[81]	UC1113 × Kofa	BP	93	5	YPC
[121]	Langdon × G18-16	RIL	152	55	GCaC, GCuC, GFeC, GKC, GMgC, GMnC, GPC, GSC, GZnC, PGC
[54]	DT695 × Strongfield	DH	185	6	GPC
[10]	Latino × Primadur	BP	121	4	YPC
[144]	UC1113 × Kofa	RIL	93	18	GPC, SV
[145]	UC1113 × Kofa	BP	93	13	F, YPC
[5]	Svevo × Ciccio	BP	120	7	YPC
[68]	Duilio × Avonlea	RIL	134	2	BG
[146]	Langdon × G18-16	RIL	152	15	GSeC, GSeY
[87]	Colosseo × Lloyd	BP	176	12	YPC
[87]	Kofa × Svevo	BP	249	4	YPC
[87]	Meridiano × Claudio	BP	181	6	YPC
[72]	Svevo × Y12-3	RIL	208	9	GPC
[147]	Saragolla × 02-5B-318	RIL	135	9	GPC
[148]	Pelissier × Strongfield	DH	162	6	SV
[149]	Worldwide elite collection	GWAS	93	20	YPC
[150]	Agrogen	GWAS	104	19	AX
[35]	Agrogen	GWAS	230	7	BG
[86]	Durum collection	GWAS	124	6	YPC
[151]	Canadian durum wheats	GWAS	169	6	YPC
[152]	Canadian durum lines	GWAS	192	28	YPC
[153]	Mediterranean landraces	GWAS	172	14	GPC, GS, TW, YPC

Abiotic stress: CC: Chlorophyll content; CIR: carbon isotope ratio; CL: coleoptile length; DB: dry biomass; FLRI: flag leaf rolling index; NDVI: normalized difference vegetation index; OP: osmotic potential; PDL: length of the ear peduncle; RRT: root-related trait; SPAD: Chlorophyll concentration measure. Biotic stress: CP: *Claviceps purpurea*; FHB: Fusarium head blight; LR: leaf rust; LS: loose smut; PM: powdery mildew; SBCMV: soil-borne cereal mosaic virus; SR: stem rust; STB: Septoria tritici blotch; YR: yellow rust. Quality: AX: Arabinoxylan; BG: β-glucan; Fb: flour yellow color; GCaC: grain calcium concentration; GCdC: grain cadmium concentration; GCuC: grain copper concentration; GFeC: grain iron concentration; GKC: grain potassium concentration; GMgC: grain magnesium concentration; GMnC: grain manganese concentration; GPC: grain protein content; GS: gluten strength; GSC: grain sulfur concentration; GSeC: grain selenium concentration; GSeY: grain selenium yield; GZnC: grain zinc concentration; PGC: phosphorus grain concentration; SV: SDS-sedimentation volume; TW: test weight; YPC: yellow pigment content.

**Table 3:** Comparison between biparental QTL mapping and genome-wide association study (GWAS).

Linkage QTL Mapping	GWAS
Two known ancestors (parents)	Multiple (unknown) ancestors
Short known recombination history	Long (unknown) recombination history
Simple population structure	Complex population structure
LD caused by linkage	LD caused by different genetic events
Requires construction of specific maps	Existing maps can be used
Contrasting genetic background	Diverse genetic background
Phenotyping is required for new populations	Phenotyping data might already be available

**Table 4:** The number of QTL per single trait and chromosome.

	Trait	1A	1B	2A	2B	3A	3B	4A	4B	5A	5B	6A	6B	7A	7B	Total
**Abiotic**	CC	1	1		1			1		1	1	1				7
	CIR							1		1	2		1		1	6
	CL		1				1		2			1				5
	DB	1			2	1			1	1	1			1	1	9
	FLRI	1		1	2				1	1	1		1		1	9
	NDVI	1	3	3	6	1	6	2	3	3	3	4	3	4	5	47
	OP			1	1	1	1		1	1	1	1	1			9
	PDL				1	1	1								1	4
	RRT	10	0	6	17	14	4		17	7	3	14	9	7	8	116
	SPAD	1	2	2	2	1	5	2	3		3		1	1	2	25
	Total	15	7	13	32	19	18	6	28	15	15	21	16	13	19	237
**Biotic**	CP		1	1						1	1					4
	FHB	1	2	7	3	3	5		2	2	4	2	4	4	3	42
	LR	6	1	7	9	4	6	4	3	5	1	4	2	4	3	59
	LS												1	1		2
	PM	1			1						1		1			4
	SBCMV			1	3	1	1		1	1			1		1	10
	SR	1	3	2	6	1	4	10	0	1	3	6	3	4	8	52
	STB	7			1	10		1		1						20
	YR		2	3	1		2	1	1		2		1	4	3	20
	Total	16	9	21	24	19	18	16	7	11	12	12	13	17	18	213
**Quality**	AX	2	2	2	1	1	1		1	3		1	1	3	1	19
	BG	1		3	2						1			2		9
	Fb							2			1	1		1	2	7
	GCaC									1	4					5
	GCuC						2	5	3							10
	GFeC			1	5	3	1									10
	GKC												8			8
	GMgC										2					2
	GMnC								2							2
	GPC	2	4	6	4	1	2	4	3	2	3	1	1	1	5	39
	GS	1					1								1	3
	GSC													1	4	5
	GSeC		2			2				1					4	9
	GseY		1		2				1	1					1	6
	GZnC			6												6
	PGC												2	1		3
	SV	2	2		2	2	3	1	1			2	1			16
	TW				1	1			1							3
	YPC	7	11	5	1		10	6	7	7	6	10	3	22	13	108
	Total	15	22	23	18	10	20	18	19	15	17	15	16	31	31	270
		46	38	57	74	48	56	40	54	41	44	48	45	61	68	720

Color gradient for the number of traits per chromosomes: from green (lower number) to red (higher number). Color gradient for the number of QTL per trait: low intensity (lower number) to high intensity (higher number). Abiotic stress (green): CC: Chlorophyll content; CIR: carbon isotope ratio; CL: coleoptile length; DB: dry biomass; FLRI: plag leaf rolling index; NDVI: normalized difference vegetation index; OP: osmotic potential; PDL: length of the ear peduncle; RRT: root-related trait; SPAD: Chlorophyll concentration measure. Biotic stress (red): CP: *Claviceps purpurea*; FHB: Fusarium head blight; LR: leaf rust; LS: loose smut; PM: powdery mildew; SBCMV: soil-borne cereal mosaic virus; SR: stem rust; STB: Septoria tritici blotch; YR: yellow rust. Quality (blue): AX: Arabinoxylan; BG: β-glucan; Fb: flour yellow color; GCaC: grain calcium concentration; GCdC: grain cadmium concentration; GCuC: grain copper concentration; GFeC: grain iron concentration; GKC: grain potassium concentration; GMgC: grain magnesium concentration; GMnC: grain manganese concentration; GPC: grain protein content; GS: gluten strength; GSC: grain sulfur concentration; GSeC: grain selenium concentration; GSeY: grain selenium yield; GZnC: grain zinc concentration; PGC: phosphorus grain concentration; SV: SDS-sedimentation volume; TW: test weight; YPC: yellow pigment content.

## Data Availability

The data present in this study is available within the manuscript and its supplementary information.

## Supplementary Materials

Supplementary materials can be found at https://www.mdpi.com/2223-7747/10/2/315/s1. Table S1: QTL database.
